# Plasticity predicts evolution in a marine alga

**DOI:** 10.1098/rspb.2014.1486

**Published:** 2014-10-22

**Authors:** C. Elisa Schaum, Sinéad Collins

**Affiliations:** Institute of Evolutionary Biology, University of Edinburgh, Ashworth Laboratories, West Mains Road, Edinburgh EH9 3JF, UK

**Keywords:** phenotypic plasticity, adaptation, *Ostreococcus tauri*, climate change, oceanography

## Abstract

Under global change, populations have four possible responses: ‘migrate, acclimate, adapt or die’ (Gienapp *et al*. 2008 Climate change and evolution: disentangling environmental and genetic response. *Mol. Ecol.* **17**, 167–178. (doi:10.1111/j.1365-294X.2007.03413.x)). The challenge is to predict how much migration, acclimatization or adaptation populations are capable of. We have previously shown that populations from more variable environments are more plastic (Schaum *et al*. 2013 Variation in plastic responses of a globally distributed picoplankton species to ocean acidification. *Nature* **3**, 298–230. (doi:10.1038/nclimate1774)), and here we use experimental evolution with a marine microbe to learn that plastic responses predict the extent of adaptation in the face of elevated partial pressure of CO_2_ (pCO_2_). Specifically, plastic populations evolve more, and plastic responses in traits other than growth can predict changes in growth in a marine microbe. The relationship between plasticity and evolution is strongest when populations evolve in fluctuating environments, which favour the evolution and maintenance of plasticity. Strikingly, plasticity predicts the extent, but not direction of phenotypic evolution. The plastic response to elevated pCO_2_ in green algae is to increase cell division rates, but the evolutionary response here is to decrease cell division rates over 400 generations until cells are dividing at the same rate their ancestors did in ambient CO_2_. Slow-growing cells have higher mitochondrial potential and withstand further environmental change better than faster growing cells. Based on this, we hypothesize that slow growth is adaptive under CO_2_ enrichment when associated with the production of higher quality daughter cells.

## 1. Introduction

Shifts in the environment drive both plastic and evolutionary responses in organisms, and theoretical studies have shown that plastic responses are good candidates for predicting evolutionary ones [1–3], but to our knowledge, no direct experimental tests of this exist. Here, we present an empirical study that tests and quantifies links between the two processes. Phenotypic plasticity is a single genotype's ability to produce variable phenotypes in response to environmental conditions [4] and views on the possible relationship between phenotypic plasticity and evolution fall into two main groups with mutually exclusive predictions. The first is that populations made up of plastic individuals are more likely to adapt to novel and changing environments [3,5–9]. This is based on population genetics models [10–12], where plasticity acts mainly by keeping population sizes high enough to maintain and/or produce variation, though it has never been tested in the absence of demographic effects. The second is that populations made up of plastic individuals are less likely to adapt [13–15]. This is possibly a result of qualitative descriptions for possible outcomes of climate change scenarios that phrase possibilities as either/or (‘migrate, acclimatize, adapt or die’), and is usually based on verbal arguments rather than mathematical models or simulations.

The misleading statement that populations respond to environmental change through *either* plasticity *or* evolution stems from two sources: first, in studies of natural populations, it is difficult to disentangle plastic and evolutionary responses, and when plastic responses cannot be ruled out, the implication is often that evolutionary responses are absent (e.g. [16,17]). While failing to rule out a plastic response does not imply that no evolutionary response is present or possible, it is harder to definitively detect an evolutionary response than a plastic one [18]. Evolutionary responses will consequently be reported less than plastic ones in proportion to the extent that they actually occur [19]. Second, a common thought experiment proposes that the environment changes such that fitness decreases, causing populations made up of individuals that can mitigate fitness loss by plasticity to be under weak or no selection. A population that is not under selection will not have to adapt. By contrast, populations with no or insufficient adaptive plastic responses will have to either migrate or adapt to avoid going extinct. This leads to the conclusion that adaptive plasticity and genetic adaptation should be negatively correlated [13,14], despite the growing body of theoretical work predicting the opposite [1–3,20]. While the relationship between adaptive plasticity and adaptation is uncontroversial within disciplines, this relationship is a source of conflicting predictions between disciplines, particularly evolutionary and marine biology, and must be empirically tested in order to estimate the extent to which plasticity data can be used to predict the evolutionary fate or adaptive potential of populations [21]. These conflicting predictions are especially important in the context of understanding how marine populations are likely to respond to global change. There are empirical studies on how large microbial populations respond to environmental perturbations in the short term through phenotypic plasticity in the absence of genetic change [22–26], or evolve in the long term using genetic change [27–32]. Despite this, neither group of studies has measured links between phenotypic plasticity and evolution.

The link between plasticity and evolution is ecologically relevant, and here we explore it in the context of marine microbes: as the world changes and oceans become less basic, more stratified and depleted in nutrients, large populations of marine microbes with short generation times will have ample scope for evolution [33]. In the context of climate change, marine biologists often base predictions on future oceans on short-term experiments [34,35], but the predictive power of plasticity data remains untested. Evolutionary biologists use models to predict, for example, how different rates of environmental change may require different levels of plasticity in order to keep fitness constant (e.g. [2]), but little is known about how the costs and benefits of plasticity affect the adaptive potential of large populations, where even a substantial drop in fitness is unlikely to lower population size to the point where natural selection cannot act effectively. Environmental fluctuations are also expected to increase in the future, which may select for increased plasticity, which could subsequently alter the speed or outcome of evolution and consequently, affect evolved phenotypes (e.g. [36,37]). The evolution of plasticity is expected when the frequency of fluctuations is on a scale of few generations relative to the organism's generation time [38]. While there are studies that characterize evolution in complex and fluctuating environments [3,39], this has yet to be applied to understanding how marine phytoplankton are likely to evolve under climate change scenarios.

Here, we use experimental evolution with a globally distributed marine picoplankton to measure how phenotypic plasticity affects evolution. We show that populations founded from more plastic ancestors evolve more, and that phenotypic plasticity in a fitness-related trait can be used as a predictor for the magnitude of an evolutionary response. We evolved 16 physiologically distinct lineages of the species-complex [40,41] *Ostreococcus* from single cells for 400 generations in constant and fluctuating environments at ambient (430 ppm CO_2_) and elevated partial pressure of CO_2_ (pCO_2_) levels (predicted for the year 2100: 1000 ppm CO_2_, based on the Intergovernmental Panel on Climate Change report 2007, [42]). We refer to the selection environments as follows: stable ambient (SA), fluctuating ambient (FA), stable high (SH) and fluctuating high (FH). The lineages varied initially in their plastic responses in oxygen evolution rates to CO_2_ enrichment [22]. We present four main findings. First, under CO_2_ enrichment, variations in plastic responses (change in oxygen evolution rates) before evolution predict evolutionary responses (change in growth rate). Second, plasticity evolves in fluctuating environments and degrades in constant ones. Third, natural selection in constant and fluctuating environments produces radically different phenotypes. Finally, lineages evolved under long-term carbon enrichment eventually grow more slowly than lineages under short-term carbon enrichment, thereby producing less but better quality daughter cells, which indicates that slow growth can be adaptive in our experimental set-up.

## 2. Material and methods

### (a) Lineages and culturing conditions

*Ostreococcus* lineages were obtained from the Roscoff Culture Collection and the Plymouth Marine Laboratory, grown in Keller Medium [43] and made clonal by dilution and propagated as described in [22]. Lineages were grown in a closed system in semi-continuous batch cultures at low densities (maximum density of 10^4^). In the selection experiment, algae were subjected to one of the following four selection regimes: selection for growth at 430 ppm CO_2_ (final average ± s.d. CO_2_: 444 ± 43 ppm CO_2_), selection for growth at 1000 ppm CO_2_ (final average ± s.d: 1031 ± 87 ppm CO_2_), selection for plasticity in an environment that fluctuated around a mean of 430 ppm CO_2_ (490 ± 97 ppm CO_2_), and selection for plasticity at high pCO_2_, in an environment where CO_2_ levels were fluctuating around a mean of 1000 ppm CO_2_ (1012 ± 244 ppm CO_2_). More precisely, in the fluctuating selection regime, pCO_2_ in the incubator was changed to a random value between 430 and 630 µatm CO_2_ once per week in the FA environment and was changed to a random value between 700 and 1300 µatm (also once per week) in the FH environment. This rate of environmental fluctuation maintains plasticity rather than multiple specialist lineages within populations (see Results). The slightly higher mean of pCO_2_ between FA and SA treatment (see the electronic supplementary material, table S1) has no overall effect (*p* = 0.42)—when we use mean and variation from mean as explaining variables in our ANOVAs, the differences between SA and FA lineages are indeed driven by differences in environmental *variability*. For assays, samples were pre-acclimatized and acclimatized for five to seven asexual generations to 430 ppm CO_2_ or 1000 ppm CO_2_ (see the electronic supplementary material, figure S1 for experimental design). The selection environments were established by setting the incubator to the appropriate pCO_2_ and by using air-stones to aerate the seawater to be used at each transfer for at least 24 h prior to transfer.

### (b) Growth rate *μ*

At *t* = 0, 100 and 400 (generations), cell count was determined using flow cytometry (FACSCalibur and FACS CANTO) and growth rate (*μ*) calculated as described in [22].

Growth rate data were used to calculate evolutionary responses, i.e. heritable differences in growth rates between populations evolved for 400 generations.

Growth responses were calculated using the following formula:
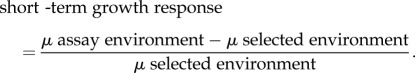
The formulae for direct and correlated responses after evolution can be found in the electronic supplementary material. Here, direct responses refer to traits of SH and FH evolved lineages measured at 1000 ppm CO_2_ relative to growth rates of SA and FH evolved lineages measured at 1000 ppm CO_2_. Correlated responses refer to traits of FH-evolved and SH-evolved lineages measured at 430 ppm CO_2_ (ancestral environment) relative to phenotypes of SA- and FA-evolved lineages measured at 430 ppm CO_2_. Unless stated otherwise, measurements were carried out after 400 generations of selection.

All populations were pre-acclimatized and acclimatized to their respective assay environment for five to seven generations each.

### (c) Flow cytometry for determination of cell density and health

FACS flow cytometry was used to determine event number (cell density), orange fluorescence, mitochondrial potential in rhodamine 123 stained cells and green fluorescence in green fluorescent protein (GFP)-modified *Ostreococcus* strains. To determine rhodamine 123 fluorescence, 1 µl of a (0.2 µg ml^−1^) rhodamine solution was added to 200 µl of sample and left to incubate for 30 min prior. Rhodamine 123 fluorescence quantifies the strength of the proton gradient across mitochondrial membranes [44] and was detected as green fluorescence.

### (d) Heat shock assay

To assess how well lineages would deal with stress, we first determined the lethal temperature threshold for our populations, and then chose a temperature that reduced viability of SA-evolved lineages by 50%. In previous trials, we had found that this is the case at a sudden 4°C increase and incubation at that temperature (22°C) for 1 h. Viability and growth rate were measured using flow cytometry over the next five to seven cell divisions.

### (e) Oxygen evolution rates

Here, we use oxygen evolution rates as an example of a plastic trait other than fitness that can be related to evolution. This requires that the trait be correlated with growth, which is indeed the case in *Ostreococcus* [22]. Net oxygen evolution and consumption were measured and used to calculate plastic responses as described in [22].

### (f) Carbonate chemistry

Seawater carbonate chemistry was calculated from pH and alkalinity using the CO2sys software [45]. Dissolved inorganic carbon (DIC) was measured colourimetrically. Total alkalinity was inferred from linear Gran-titration plots (see the electronic supplementary material, table S1). DIC and pH samples for all lineages were taken at the beginning and at the end of the selection experiment. Additionally, we measured DIC and pH or alkalinity prior to using the bubbled medium in a transfer.

### (g) Green fluorescent protein (GFP) strains/competition assay

To test the hypothesis that slow growers are better competitors [46], we competed eight representative lineages against a GFP lineage (transformed oth95) [40]. For the competition experiment, 20 ml of medium were inoculated with 100 µl of wild-type lineages and GFP populations, and cell numbers for each were recorded every day for a 14 day period (two transfers). Each lineage's competitive ability was calculated relative to the GFP-modified lineages (as fold difference in growth) and plotted as a function of the lineage's growth rate in single culture.

### (h) Clones/composition of evolved populations

In order to assess whether populations were composed of plastic lineages or of a mixture of non-plastic lineages, bio-replicates of a subset of seven representative lineages were made clonal by dilution, and growth rates were then determined for at least three clones per lineage.

### (i) Data analysis

Data were analysed in the R environment, using linear mixed effects models in the nlme and lme4 packages. Data were tested for normality and heterogeneity prior to performing ANOVAs on the models with selection regime and assay environment as fixed effects. Depending on the test performed, there were up to three random effects per model that were all treated as un-nested.

## 3. Results

### (a) Plasticity predicts the extent of evolution

**Figure 1.**
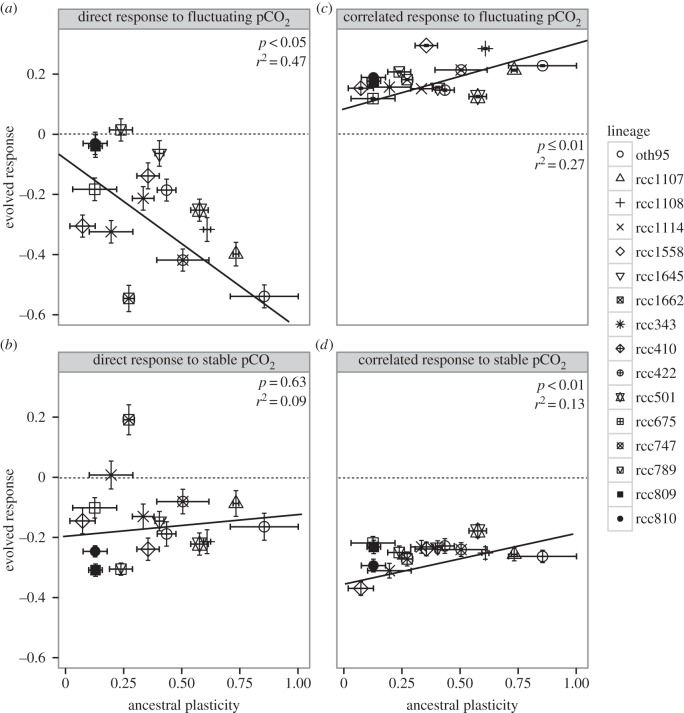
(*a*–*d*) Lineages with higher ancestral plasticity evolve more. Direct and correlated responses to selection plotted as a function of plasticity in oxygen evolution rates before evolution (ancestral plasticity). For all panels (*a*–*d*), different shapes represent mean values for each lineages ± 1 s.e. For each lineage *n* = 3. Dashed line indicates no response to selection. Panel (*a*) (selection in FH, assay at 1000 ppm CO_2_): ancestral plasticity in FH evolved lineages predicts up to 47% of the evolutionary response (*F*_2,13_ = 210.67, *p* < 0.001). FH populations evolve slow growth in response to high pCO_2_. Panel (*b*) (selection in SH, assay at 1000 ppm CO_2_): with no selection for plasticity, a linear relationship using ancestral plasticity as the only explaining variable is not statistically significant (*p* = 0.63). Still, most lineages evolve lower growth rates (range from −0.31 to −0.08, mean −0.15 ± 0.12). Panel (*c*) (selection in FH, assay at 430 ppm CO_2_): ancestral plasticity is a significant nonlinear predictor of the correlated response to selection (*F*_2,13_ = 563.38, *p* < 0.0001). Lineages from FH increased their growth rate at ambient pCO_2_ the most when their ancestral plasticity was high (increase in growth of 0.12–0.30, mean 0.19 ± 0.05). Panel (*d*) (selection in SH, assay at 430 ppm CO_2_): lineages selected in SH had a negative correlated response, and the relationship between ancestral plasticity and the correlated response to selection was significant (*F*_2,13_ = 22.28, *p* < 0.01), though best described by a nonlinear fit (*p*-values and *r*^2^ reported on the panels are for linear regression).

The main question that our experiment was designed to answer is how ancestral plasticity, measured as change in oxygen evolution rates to in response to elevated pCO_2_ prior to selection (at *t* = 0), relates to evolution (heritable changes in growth rate) within a lineage. The direct response to selection for growth at high pCO_2_ is calculated by comparing the growth rates in high pCO_2_ of populations evolved at high pCO_2_ (here, FH or SH) with the growth rates in high pCO_2_ of populations evolved in ambient CO_2_ (here, FA or SA). We find that *Ostreococcus* evolves in response to CO_2_ enrichment (figure 1; *F*_3,132_ = 155.66, *p* < 0.0001), and populations with more plastic ancestors evolve more in high pCO_2_ than populations founded from less plastic ancestors (*F*_3,132_ = 55.90, *p* < 0.05). In FH lineages, ancestral plasticity explains almost half of the direct response to selection (figure 1*a*; *F*_1,120_ = 167.66, *p* < 0.001) and 20% of the correlated response to selection (*F*_1,120_ = 238.77, *p* < 0.0001). By contrast, clade/species explains 5–15% of the variation in evolutionary responses in FH populations. Lineages selected in the stable SH environment also evolve (figure 1*b*; *F*_1,120_ = 122.27, *p* < 0.001 and figure 1*d*; *F*_1,120_ = 593.50 *p* < 0.0001 for direct and correlated responses, respectively), but the relationship between ancestral plasticity and evolution is weaker, and trends in the opposite direction, perhaps because plasticity is not under selection in constant environments.

**Figure 2.**
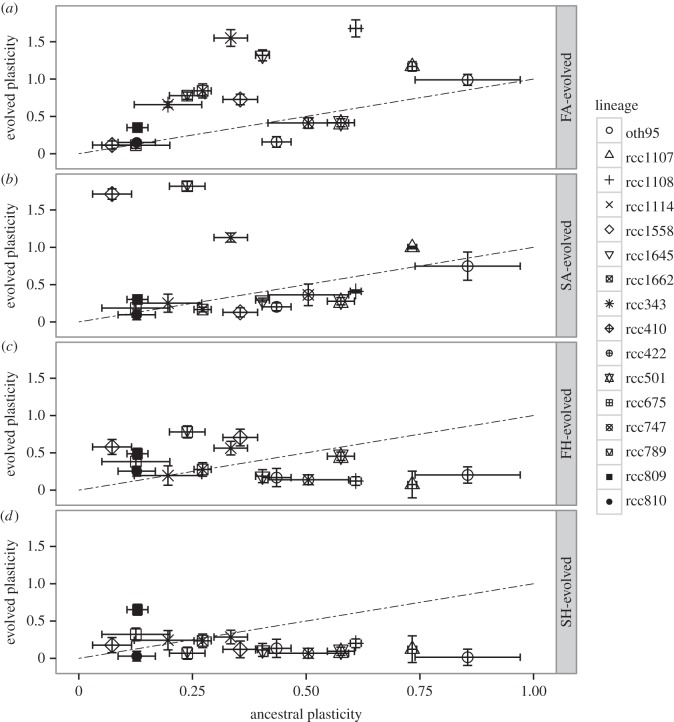
Plasticity evolves in fluctuating environments. Plasticity in oxygen evolution rates in four selection regimes at the beginning at the selection experiment (*x*-axis) differs from plasticity in oxygen evolution rates after 400 generations of selection (*y*-axis). The dotted 1 : 1 line indicates no change in plasticity after 400 generations of evolution. Each symbol represents mean plasticity ± 1 s.e. for a lineage, with three replicate populations per lineage. Panel (*a*) (FA regime): plasticity changes significantly (*F*_1,98_ = 5.58, *p* < 0.05) and increases in 69% of lineages. Panel (*b*) (SA regime): in all but four lineages, plasticity does not change significantly. In the four lineages where plasticity does change, it increases (post hoc *p* < 0.001). Panel (*c*) (FH regime): 56% of all populations evolve higher plasticity (*F*_1,98_ = 6.01, *p* < 0.05) after selection at FH. Panel (*d*) (SH regime): plasticity increases in four out of 16 lineages, but decreases or remains unchanged in all other lineages (but see the electronic supplementary material, figure S3).

**Figure 3.**
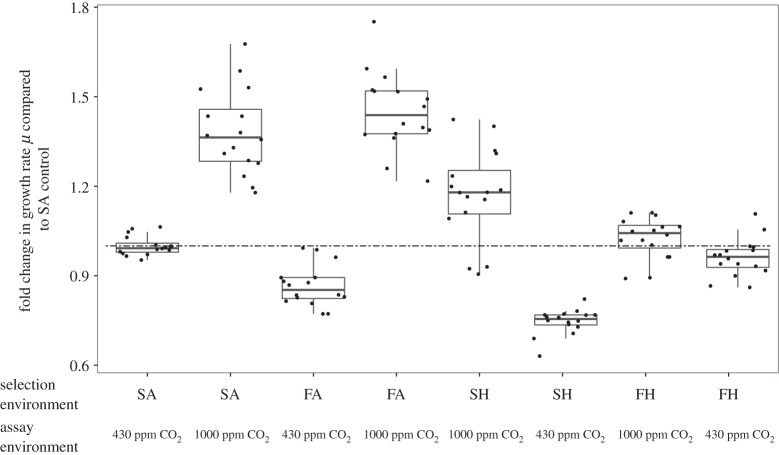
Evolution at elevated pCO_2_ reverses short-term growth responses to changes in pCO_2_. Selection regime changes how lineages respond to CO_2_ enrichment following 400 generations of selection (*F*_7,105_ = 123.38, *p* < 0.001). Here, *n* = 48 for each selection and each assay environment. The dashed line indicates no change compared with SA populations. Populations selected in SA show the expected plastic response in growth to CO_2_ enrichment after 400 generations of selection, where mean growth rate at elevated pCO_2_ is higher than at 430 ppm CO_2_ (*F*_7,105_ = 114.88, *p* < 0.001, mean growth rate of SA populations assayed at 430 ppm: 0.67 ± 0.02 d^−1^, mean growth rate of SA population assayed at 1000 ppm CO_2_: 0.79 ± 0.03 d^−1^). When grown at elevated pCO_2_, the growth rate of lineages selected at FH or SH is lower than the short-term response of their respective controls (i.e. FA or SA) growing at high pCO_2_ (post-hoc FH *p* < 0.05 and SH *p* < 0.05). Populations selected at SH also have negative correlated responses to selection. Growth rate of SH lineages at 430 ppm CO_2_: 0.5 ± 0.01 d^−1^, after growth rates have recovered, while lineages selected in FH have an average growth rate of 0.69 ± 0.02 d^−1^.

The correlated response to selection for growth at high pCO_2_ is calculated by comparing the growth rates in ambient pCO_2_ of populations evolved at high pCO_2_ (here, FH or SH) with the growth rates in ambient pCO_2_ of populations evolved in ambient pCO_2_ (here, FA or SA). The correlated response to selection in SH shows a strong nonlinear correlation between ancestral plasticity and evolution, where low-to-medium ancestral plasticity correlates with an increase in the evolutionary response, but where there is no further increase in the correlated evolutionary response for ancestral plasticity over 0.3. Plasticity degraded (figure 2) in SH evolved lineages, and these lineages display arrested growth (figure 3) in ambient pCO_2_.

### (b) Plasticity evolves or is maintained in fluctuating environments

Fluctuating environments select for plasticity in our experiment. Growth for 400 generations in fluctuating environments yields populations composed of individuals that are more plastic than their ancestors (figure 2*a,c*; also see the electronic supplementary material, figure S2). Plasticity changes significantly in the FA selection environment, where selection was for plasticity alone (figure 2*a*, *F*_1,98_ = 5.58, *p* < 0.05). There, it increases in 11 of 16 lineages, remains unchanged in two, and is reduced in three out of all 16 lineages (average fold increase 1.69 ± 0.39, post hoc *p* < 0.05). In the FH environment, where populations were selected for both plasticity and growth at high pCO_2_, evolved plasticity after 400 generations of selection is on average higher than ancestral plasticity (*F*_1,98_ = 6.01, *p* < 0.05), with the average increase in plasticity being 1.67 ± 0.51-fold (post hoc *p* < 0.05).

Stable environments do not select for plasticity here, and plasticity does not change significantly after 400 generations of evolution in stable environments. In the SA environment (*F*_1,98_ = 0.16493, *p* = 0.2), it remains unchanged in 12 lineages and increases significantly in four. Plasticity decreases over time in 10 of 16 SH lineages (*F*_1,98_ = 10.6, *p* < 0.05). However, this apparent overall decrease in plasticity may be due to arrested growth in the ancestral environment in some SH lineages (no growth means we cannot quantify plasticity; see the electronic supplementary material, figure S3).

After 400 generations, we find a tendency in all selection regimes for plasticity to decrease in lineages with the highest ancestral plasticity (average 0.86-fold ± 0.44 change, also electronic supplementary material, figure S4) and to increase in most other lineages (average change 1.69-fold ± 0.39).

### (c) Evolution reverses the plastic response to CO_2_ enrichment

Populations evolved in the ambient CO_2_ environments, SA and FA, respond to short-term increases in CO_2_ by increasing their growth rates (*F*_7,105_ = 114.88, *p* < 0.001; figure 3). Increasing growth is the usual short-term response of *Ostreococcus* to CO_2_ enrichment [22]. However, after about 100 generations (electronic supplementary material, figure S5), populations selected in high pCO_2_ environments (SH and FH) decrease their growth rates at high pCO_2_ relative to both their own ancestors and to populations selected at ambient pCO_2_. SH- and FH-selected populations eventually and completely reverse the plastic response to high pCO_2_ and return to growth rates at high pCO_2_ that are similar to SA-selected populations growing at 430 ppm CO_2_, showing the evolution of slow growth during an experiment where the culturing method should select for rapid growth.

After 400 generations of selection in elevated pCO_2_ environments, responses to ambient CO_2_ have changed. FH lineages grow better in their ancestral environment of 430 ppm CO_2_ (correlated response) than do SH lineages. When SH lineages are transferred back to 430 ppm CO_2_, they do not grow detectably for about two weeks. By contrast, FH lineages do not show arrested growth.

### (d) Slow-growing cells are better at competing, withstanding heat shock and maintaining mitochondrial potential than fast-growing cells

FH-selected populations have lower growth rates than SH-selected populations at high pCO_2_, which would reflect a cost of plasticity of 14–18%, though we argue in the discussion that this interpretation is probably incorrect, or at least a misleading oversimplification, as slow growers are better competitors, better able to withstand heat shock, and have higher mitochondrial potential than lineages with chronically elevated growth rates.

In populations that had evolved to grow slow (FH) or been selected in environments that did not increase growth initially (SA, FA), lineages with low growth rates in monoculture had better competitive abilities than lineages with high growth rates in monoculture (*F*_1,177_ = 810.61, *p* < 0.0001; electronic supplementary material, figure S6). In SH-evolved populations however, lineages in monoculture with low growth rates were worse competitors in mixed culture—though SH lineages overall grew quickly and were also all poor competitors compared to lineages evolved in other environments. A similar pattern was observed in a study using *Chlamydomonas* [46]. If low growth rates in monoculture reflected a cost of plasticity, it would be expected that populations evolved in SH be better competitors than those evolved in FH. Instead, populations evolved in FH grow more slowly in monoculture but are in fact better competitors in mixed culture than populations evolved in SH (electronic supplementary material, figure S7).

We hypothesized that slow-growing cells were better competitors because they produced better quality daughter cells than did fast-growing cells. We tested this by measuring two indicators of cellular health. First, elevated levels of orange fluorescence have been shown to increase in stressed or moribund algae [47]. We find that levels of orange fluorescence at the end of the experiment are up to 20 times higher in cells from SH populations than in cells from SA populations or fluctuating populations, indicating that non-plastic populations are more stressed than plastic populations when grown under chronic high pCO_2_ conditions. Second, mitochondrial potential is higher in the slower growing lineages from the FH environment than the faster growing SH-selected lineages (*F*_3,109_ = 15.74, *p* < 0.05; see the electronic supplementary material, figure S8). In addition to appearing less healthy when alive, fast-growing cells are less likely to survive heat shock. FH-selected lineages have lower growth at high pCO_2_, but higher viability and growth rates after heat shock than lineages selected in SH, indicating that they are better able to withstand stress than fast-growing lineages (*F*_1,236_ = 9.52, *p* < 0.005 and *F*_1,236_ = 53.27, *p* < 0.0001, for viability and growth rates, respectively; electronic supplementary material, figure S9). This supports our hypothesis that a greater reduction in growth rate in populations evolved under chronic CO_2_ enrichment correlates with the production of better quality cells.

## 4. Discussion

### (a) Plasticity predicts evolution

We have shown that, in a fluctuating environment, ancestral plasticity can explain almost half of the direct response to selection (figure 1), and that *Ostreococcus* lineages founded from more plastic ancestors evolve more in high pCO_2_ environments than lineages founded from less plastic ancestors. This makes ancestral plasticity a good predictor of eventual evolutionary responses and supports theoretical studies arguing that plasticity should facilitate evolution [1–3,20]. Most explanations on why adaptive plasticity facilitates evolution focuses on the effects of differences in population sizes between populations made up of plastic and non-plastic individuals [3,20,48]. Here, there is no systematic difference in population size between treatments (see the electronic supplementary material). Our results show that even in the absence of demographic effects that would affect the amount of genetic variation possible in the population, plasticity can facilitate evolution. This is consistent with plasticity affecting the phenotypic and fitness effects of mutations directly [1]. Interestingly, this suggests that individual plasticity in large microbial populations may be maintained partially as a by-product of more plastic types being more able to adapt, and thus being less likely to go extinct, than less plastic types, and may partly explain why microbes that can respond to environmental change genetically also maintain high levels of individual plasticity.

### (b) Plasticity evolves or is maintained in fluctuating environments

Depending on its rate and predictability, environmental variation can select either for the evolution of plastic individuals [3], of generalists with invariable phenotypes over several environments [49,50], or of communities made up of many specialists [49,51,52]. Here, we found an overall increase of individual plasticity in lineages selected in fluctuating environments. We also observe that the lineages with the highest ancestral plasticity evolve slightly lower plasticity. This could be caused by lineages decreasing plasticity enough to limit its cost, while still remaining plastic enough to persist in a fluctuating environment, a strategy known as phenotypic buffering [53,54]. There may be some optimum amount of trait plasticity for the rate and magnitude of change in fluctuating environments used in this experiment, and populations may be converging on that—in the high pCO_2_ selection environments variance of evolved plasticity is almost half that of ancestral plasticity (see the electronic supplementary material, table S2). Here, an optimal level of plasticity is more likely than a reduction in plasticity that is due to costs associated with higher levels of plasticity.

Additionally, evolutionary history may limit the ability to evolve plasticity, since none of the ‘deep-sea’ lineages of *Ostreococcus* display any significant change in plasticity during the evolution experiment; these lineages were isolated from relatively constant environments and had lower ancestral plasticity than surface strains [22]. However, among the surface strains, variation in plasticity is the best predictor of variation in evolutionary responses.

We have discussed heritable changes as mutations, but these could be a combination of genetic and epigenetic contributions, if epigenetic changes are stable for at least 14 generations (the time used for acclimatization) or are encoded by genetic mutations.

### (c) Evolution reverses the plastic response to CO_2_ enrichment

*Ostreococcus* evolves in response to selection at elevated pCO_2_. After 400 generations of selection at elevated pCO_2_, lineages selected in the SH environment fail to grow in their ancestral environment (SA). This pattern has also been reported in fresh water green algae [31] and coccolithophores [28,29]. By contrast, FH-selected populations do not show arrested growth at 430 ppm CO_2_. These results are consistent with the maintenance or the evolution of plasticity in fluctuating environments. Under climate change scenarios, there may be different modes of what selection acts on, favouring either phenotypic plasticity where phenotype changes in response to environmental fluctuations, phenotypic buffering [53,54] or a combination of both: as described in [22,55], in the short-term, CO_2_ enrichment may be beneficial to the most plastic lineages that are best at taking advantage of the new situation. More plastic lineages will be selected for in the short-term, and adaptive evolution will occur through lineage sorting of these lineages. In the long-term, the new environment may cause stress at the limit of tolerance levels [56], so lineages are now selected for maintaining cellular functions and metabolic capacities, rather than how well suited they are for outgrowing other lineages in the matter of a few generations. So long as a lower growth rate does not result in immediate competitive exclusion by being overgrown, the benefit of producing higher quality daughter cells less likely to die or fail to divide has the potential to outweigh the cost of producing fewer cells. We have shown that slowed growth evolves repeatedly under our high CO_2_ culture conditions. Further experiments are needed to ascertain how frequently slowed growth evolves under different enrichment scenarios.

Green algae (including *Ostreococcus*) and cyanobacteria usually respond to short-term increases in pCO_2_ by increasing their growth rates [22,31,57,58]. Unexpectedly, growth rates then slow back down after a few hundred generations of growth at elevated pCO_2_ in our experiment. This contrasts with selection experiments in other non-calcifying and non-silicifying algae, where plastic responses to constant high pCO_2_ are maintained during evolution for up to 1000 generations [30,31,59]. It is unusual (but not unprecedented—see [60]) for growth rates to decrease in a laboratory selection experiment using large populations of microbes, especially in semi-continuous cultures that do not reach carrying capacity, which selects for rapid growth [61]. We suggest that the slow growth rates measured here are adaptive in environments with chronically elevated pCO_2_. The evolution of slow growth has previously been described in [60]. There, however, slow growth was reported to be a consequence of, rather than an avoidance strategy for, damage in ageing cells. The phenomena described are similar but the causes are not. We argue that slower growth rates seen in FH are adaptive and reflect a benefit, not a cost, of plasticity and provide more detail of our reasoning below.

Our data indicate that slowing growth is associated with higher mitochondrial function. We see that fast-growing cells have lower mitochondrial potential than slow-growing cells, which is consistent with rapid growth causing more oxidative damage than slower growth [60,62]. This may be particularly important here, since O*streococcu*s, has only a single mitochondrion [40]. We hypothesize that lower mitochondrial function may decrease fitness more in cells with only one mitochondrion than in cells with multiple mitochondria. In cells with multiple mitochondria, healthy mitochondria may have enough function to make up for the damaged ones, or, malfunctioning mitochondria may not be passed on to daughter cells, leading to no change in mitochondrial potential across generations [63,64]. In *Chlamydomonas rheinhardtii*, elevated pCO_2_ has been found to affect mitochondrial size and potential, with high pCO_2_-evolved cells shown to have smaller, more efficient mitochondria than in cells evolved in control (there, current ambient) levels of CO_2_ [65]. In organisms with one mitochondrion per cell, neither of these strategies can be applied.

The lower mitochondrial potential we find in the faster growing lineages may explain the difference in outcomes between selection experiments in *Ostreococcus*, where ancestral increases in growth under CO_2_ enrichment are reversed after several hundred generations of growth in high pCO_2_, and other algae, where growth remains high under similar conditions [30,31,59]. In addition to having cells with higher mitochondrial potentials, populations selected in fluctuating environments also survive heat shock better than populations selected in constant environments.

Taken together, these supporting results show that chronic growth at elevated pCO_2_ is stressful for *Ostreococcus* and that lower growth rates are associated with lowering that stress. This, along with the high relative fitness of slower growing lineages, supports our interpretation that slower cell division rates during evolution at high pCO_2_ are adaptive in *Ostreococcus*. In addition, plastic lineages decrease their growth rates most, indicating a benefit, not a cost, of plasticity.

## 5. Conclusion

Plastic responses can predict the magnitude of evolutionary responses, and this relationship between plasticity and evolution offers a pragmatic solution to predicting which phytoplankton populations are likely to evolve more under global change. Here, adaptation reverses plastic responses to CO_2_ enrichment and leads to the evolution of slow growth rates. In similar systems, short-term responses will overestimate changes in growth rate and trait values between contemporary and future phytoplankton populations, while underestimating genetic changes and the organisms' ability to adapt. More generally, our data suggest that since most laboratory evolution experiments so far have been carried out in constant environments even though marine environments can be highly variable, large microbial populations are more likely to adapt to ocean acidification than previously thought. This is especially relevant given predictions that both the magnitude and frequency of changes in pCO_2_ in oceans will increase in the future [66]. Our study predicts that first, large populations which are more plastic now will evolve more under global change, second, that most large populations will evolve to become more plastic in the future and third, that plastic responses which drastically increase growth rates can be reversed by natural selection because of the stress associated with maintaining rapid growth.

## Supplementary Material

Grow Slow - Supporting Information

## Data accessibility

Data will be available at https://datadryad.org/handle/10255/3/submit?workspaceID=93761.
