# Supplementary material for: Plasticity predicts evolution in a marine alga
Source: Proc Biol Sci. 2014 Oct 22;281(1793):20141486. doi: 10.1098/rspb.2014.1486 (PMC4173685; doi:10.1098/rspb.2014.1486)
Supplement: Grow Slow - Supporting Information [file rspb20141486supp1.docx]

**GROW SLOW**

**SUPPORTING INFORMATION**

**FIGURES**

**SI Figure 1: General experimental set-up** of selection experiment.. 3 biological replicates of 16 *Ostreococcus* lineages were grown in four selection environments, SA, FA, SH, and FH. Growth rates and oxygen evolution rates relevant to this chapter were assessed at the beginning of the experiment, and at the end of the experiment. At the end of the experiment growth rates and oxygen evolution rates of all lineages were measured at 430ppm CO_2_ and at 1000ppm CO_2_, to yield control values, evolved plasticity, correlated responses and direct responses. Samples for carbonate chemistry were taken and optical density determined at each transfer.

**SI Figure S2: Evolved populations are composed of plastic individuals rather than a mixture of specialists that were being maintained by fluctuating selection.** After 400 generations of selection 3 bio-replicates of 7 representative lineages (“ecotype” in figure legend) were made clonal by dilution, and their growth rates measured in the mean environment they had been selected in, as well as in the correlated environment. Variance within one lineage is very low, showing that we have indeed selected for plasticity rather than a mixture of specialists. Each symbol represents a lineage (mean ± variance), ticks on x-axes are also per lineage.

**SI Figure S3 Plasticity evolves, particularly in fluctuating environments, but lineages selected in SH do poorly in the assay environment, which affects the measurement of evolved plasticity (if cells do not grow, we cannot measure them):**

Initially, more than 60 % of lineages in the non-fluctuating selection environment display decreased plasticity. It is worth noting, that all these populations were grow very poorly upon transfer to their ancestral environment. When given a month to recover in their ancestral environment, the plastic response changes drastically. We assume that the “real” change in plasticity would have been larger than upon acclimation to the ancestral environment, but smaller than after full recovery. The acclimation response to the ancestral environment is ecologically more relevant, as under more natural conditions a poorly growing population would not be given a month to recover, but would be quickly outcompeted by other populations. Each lineage is represented by a unique symbol ± 1 standard error (n=3 bioreplicates per lineage in each selection regime).

**SI Figure S4: Oxygen evolution levels change depending on selection/assay regime (upper panel) and plasticity in oxygen evolution evolves depending on lineage and selection (lower panel):** We have used oxygen evolution as a plastic trait. Oxygen evolution rates increase (upper panel) in the short-term in response to elevated pCO_2_ for populations in both fluctuating and non-fluctuating selection regimes (F _1, 256_= 62.14 p < 0.0001). 400 generations later, some lineages still had significantly higher oxygen evolution rates than the same lineages at control conditions, but on average, oxygen evolution rates had dropped and become more similar to those at 430pmm CO_2_. When we use this dataset to calculate how much plasticity in oxygen evolution changes throughout selection (lower panel), we find that lineages are significantly different in how much plasticity they evolve (F_15,215_ = 169.16, p < 0.0001 ) and that significantly more lineages from fluctuating environments than stable environments evolve higher plasticity compared to their ancestral plasticity values. In the upper panel, the dotted line indicates mean control oxygen evolution rates. Values larger than one indicate increased oxygen evolution rates; values lower than one indicate decreased oxygen evolution rates. Black dots indicate individual lineages – no error-bars were added to lineages for clarity. Box plots were drawn with the thick band indicating the median (i.e. 2^nd^ quartile). Bar plots are ± 1 standard error. Ancestral plasticity is referred to as “initial plasticity” in the figure legend.

**SI Figure 5: over the course of 400 generations of selection, growth rate changes in response to carbon enrichment.**

Growth rates were estimated weekly just before a transfer and were calculated from optical density readings. Note that these estimates of growth may vary slightly from the more detailed growth rate analysis that was carried out using a flow cytometer at the beginning, middle and end of the experiment. Displayed are pooled growth rates for all lineages. In the beginning, lineages react to carbon enrichment by growing faster, but after less than 200 generations, growth slows down and eventually returns almost to ancestral levels of growth. Lineages in both the fluctuating and non-fluctuating environment also have slightly lower growth ends after 400 generations of selection, but this is not significant (p = 0.61).

**SI Figure 6: There is a trade-off between growth in single culture and growth in mixed culture for lineages that had been selected under control or fluctuating conditions, but not for lineages selected at stable elevated CO_2_ levels**

When grown in mixed culture with an acclimated, GFP-modified *O. tauri* lineage Oth95, lineages with comparatively high growth rates in single culture are less likely to outcompete the GFP modified strain (F _1,177_ = 810.61, p <0.0001). Notably, in the non-fluctuating elevated CO_2_ environment, the trade-off is reversed and not significant (F _1,39_ = 0.13,p= 0.72, r^2^ = 0.002). For the other selection regimes, for growth rates larger than 0.5, the trade-off is significant with F _1,39_ = 314.89, p < 0.0001, r^2^ = 0.86, F 1,39 = 312.87, p <0.0001, r^2^ = 0.86, and F _1,39_ = 217.70, p <0.0001, r^2^ = 0.82 for selection at SA, FA, and FH respectively. We conducted the experiment on a subset of eight lineages, each of which are represented by a unique symbol ± 1 SE.

**SI Figure 7: Lineages with higher evolved plasticity are better competitors:**

The overall trend is for lineages with higher evolved plasticity to be better competitors (F _1,177_ = 7.35, p < 0.05). When broken down into differnt selection regimes, we find that this correlation is only statistically significant for lineages selected in fluctuating environments, and that the correlation is in fact reversed for lineages selected at stable elevated pCO_2_. r^2^ and p – values for the control SA, FA, SH and FH are (in the same order):

r^2^= 0.31, p=0.1; r^2^=0.68, p<0.05; r^2^=-0.16, p= 0.86 and r^2^= 38, p <0.05

Each symbol represents a lineage’s mean (with n=3) ± 1 SE. We used a subset of eight representative lineages for this experiment.

**
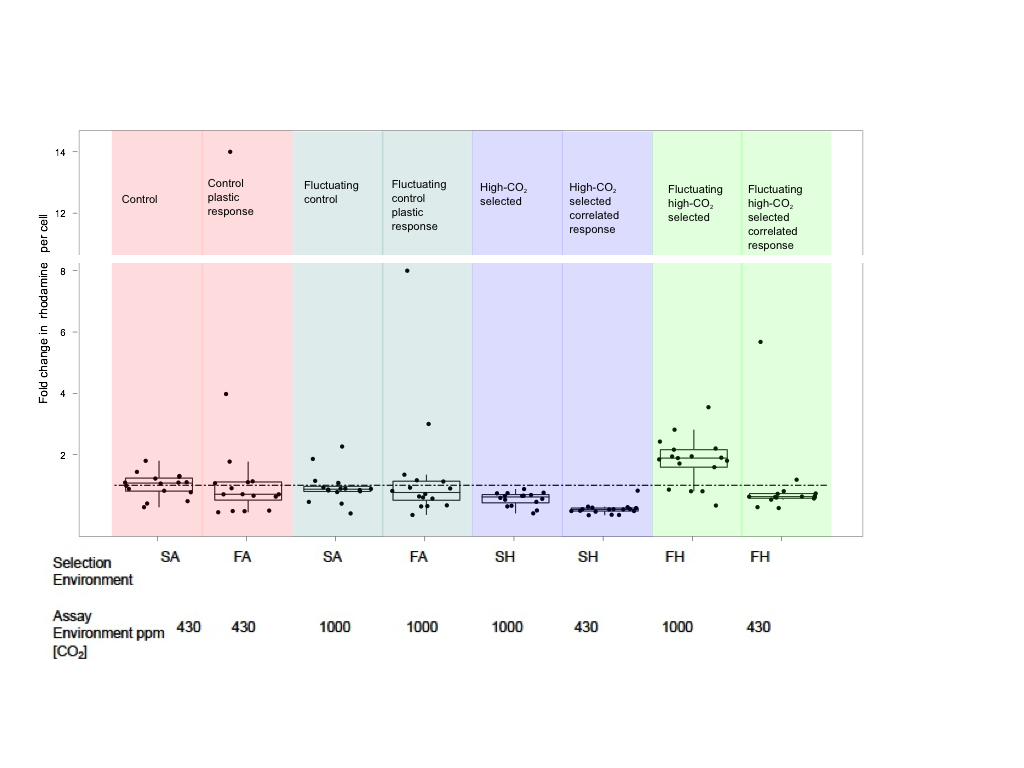
**

**SI Figure 8: Rhodamine stain 123 – there is a trend for rhodamine fluorescence to be higher in lineages from fluctuating selection regimes, indicating a higher, mores stable proton gradient across the mitochondrial membrane, which is necessary for ATP production.**

There is a significant overall difference of fluorescence across selection regimes/assays (F _3,105_ = 57.62, p <0.0001), and a post-hoc test shows that rhodamine123 fluorescence is lower than at control conditions in both assays for the 1000ppm selection regime (fold difference = 0.55 ± 0.39, p < 0.001 for the correlated response, and fold difference = 0.89 ± 0.05, p < 0.05 for the direct response), indicating decreased cellular health at stable elevated CO_2_ levels. Mean fluorescence is also slightly lower than control levels for the correlated assay in the 1000ppm CO_2_ fluctuating regime, but this is not significant (p=0.98) and at a fold change of 0.94 ± 0.13, fluorescence is still higher than in the correlated response in the non-fluctuating regime (p < 0.0001). When rhodamine123 fluorescence is measured for the direct response to selection at fluctuating elevated CO_2_ levels, there is a significant increase (fold 2.1 ± 0.12, p <0.001). In all other assays, while there is no overall difference to the control, there is a marked increase for rhodamine fluorescence in some lineages, most notably Mediterranean surface strain rcc1108, in the plastic response to carbon enrichment. All 16 lineages (3 bioreplicates) were used for this experiment, and are represented by black filled circles, of which errorbars have been omitted for clarity. Boxplots are displayed as is standard, with the thick band indicating the median. The dotted line indicates a fold change of 1, i.e. no change at all. Values below the dotted line indicate a decrease in rhodamine fluorescence compared to control values. Values above the line indicate an increase in fluorescence.

**SI Figure 9: Selection regime has a strong impact on how sensitive lineages react to a short-term 4ºC increase in temperature. Shown are overall viability (upper panel) and growth rates (lower panel) of the surviving populations**

**Upper panel**: Viability after a short-term “heat shock” is significantly different between selection regimes and assays (F_4, 236_ = 53.27, p <0.0001). Lineages from fluctuating selection regimes have overall higher viability than lineages from stable selection regimes (F_1, 236_ = 9.52, p < 0.005). For the controls, viability after being exposed to elevated temperatures is at about 50% in both the stable and the fluctuating treatment. Lineages selected in SA and FA have a viability of almost 100% when assayed at 1000ppm CO_2_, indicating that CO_2_ is in fact a nutrient that can be beneficial in the short-term. In both the direct and correlated responses to SH and FH selection, mean viability in lineages from the fluctuating selection regime is about 1.3 fold higher. Yellow boxes represent lineages from stable selection regimes, green ones, lineages from fluctuating selection regimes. Black dots are means for individual lineages. The boxplot was drawn with the thick band indicating the median. Error-bars on dots were omitted for clarity.

**Lower panel:** A temperature increase of 4ºC significantly alters growth rates across all selection regimes and assays (F _1,239_ = 44.91, p < 0.0001), generally leading to a reduction in growth rates, apart from control lineages in the 1000ppm CO_2_ assay, were there is a trend for growth rates to be slightly elevated. Growth rates µ are, on average, higher in lineages from fluctuating selection regimes than in those from stable selection regimes (F_1,236_ = 53.27, p < 0.0001). The upper dotted red line indicates the mean growth rate for control lineages at 430 ppm CO_2_, and the lower dotted red line represents the mean growth rate for “heat-shocked” control lineages. Orange bars represent data for lineages that had not been subjected to elevated temperatures. Blue bars represent data for lineages after they had been subjected to elevated temperatures. All data are ± 1 SE.

**SI Figure 11: Levels of CO_2_ [ppm] schematic plan (left) and obtained (right) in the four selection regimes used throughout the selection experiment (see table 1 for detailed carbonate chemistry):** In the control treatment (red), we aimed for a stable 430ppm CO_2_, and reached, averaged over the course of the experiment 444 ± 43 ppm CO2. For the fluctuating control (turquoise), we aimed for a mean slightly above 430 ppm CO_2_, as due to the incubator’s properties, pCO_2_ levels could not be set to be at values lower than 430 ppm CO_2_ for long periods of time. Fluctuations were chosen to be a random value between 400 ppm CO_2_ and 600 ppm CO_2_. The mean pCO_2_ reached was 490 ± 96 ppm CO_2_. In the elevated CO_2_ regime (purple), pCO_2_ levels rose from 430 ppm CO_2_ to 1000 ppm CO_2_ over the course of 3 weeks and were then aimed to remain stable at 1000ppm CO_2_, with the overall average obtained 1031 ± 87 ppm CO_2_. For selection at fluctuating elevated pCO_2_ levels, pCO_2_ was also kept below 1000ppm CO_2_ for the first 3 weeks and then aimed to fluctuate weekly around a mean of 1000ppm CO_2_. The average pCO_2_ level reached was 1012 ± 244 ppm CO_2_. Fluctuations were chosen to be a random value between 700 ppm CO_2_ and 1300 CO_2_ ppm. Differences between individual samples within the same treatment were small with an average of 7.6 ppm, 15 ppm, 16 ppm and 10 ppm CO_2_ the control, control fluctuating, elevated and elevated fluctuating selection regime respectively.

**Tables:**

**SI Table 1:**

Carbonate Chemistry: pH and DIC were measured routinely, total Alkalinity (TA) was measured at the beginning, 100 generations into the experiment and at the end of the selection experiment. All other values were calculated using seacarb within R for 18 ºC and salinity 32. pH was determined for each sample at the end of a transfer. DIC samples were taken for a subset of samples at each transfer, and for all samples at the beginning and the end of the selection experiment. Data for each time point in each selection regime was calculated from a minimum of six samples.

| **Treatment** | **Week** | **pCO2 aimed for**  **[ppm]** | **pCO2 obtained**  **[ppm]** | **pH** | **HCO_3_^-^**  **[µmol kg^-1^]** | **CO_3_^2-^**  **[µmol kg^-1^]** | **DIC**  **[µmol kg^-1^]** | **TA**  **[µmol kg^-1^]** |
| --- | --- | --- | --- | --- | --- | --- | --- | --- |
| Control | 1  2  3  4  5  6  7  8  9  10  11  12  13  14  15  16  17  18  19  20  21  22  23  24  25  26  27  28  29  30  31 | 430  430  430  430  430  430  430  430  430  430  430  430  430  430  430  430  430  430  430  430  430  430  430  430  430  430  430  430  430  430  430 | 477.22  426.43  466.76  478.44  477.79  431.15  367.72  462.38  455.04  339.35  455.43  373.96  343.32  475.92  484.24  343.91  458.27  464.11  446.81  448.00  466.14  474.71  479.70  465.74  460.06  447.83  471.33  447.92  456.82  460.06  459.08 | 8.001  8.030  8.013  8.003  8.003  8.021  8.101  8.016  8.023  8.130  8.022  8.029  8.111  8.000  8.001  8.101  8.018  8.002  8.013  8.001  8.003  8.001  8.001  8.012  8.001  8.001  8.002  8.003  8.003  8.001  8.003 | 1990.48  1900.95  1999.06  2003.79  2001.57  1880.38  1929.99  1996.29  1993.73  1904.46  1993.62  1664.34  1843.84  1980.08  2020.15  1805.47  1987.19  1939.24  1913.59  1867.78  1952.80  1980.46  2001.17  1992.99  1919.23  1867.88  1969.50  1876.06  1913.27  1919.22  1922.72 | 154.88  158.09  159.72  156.56  156.42  152.99  188.97  160.78  162.96  199.39  162.80  138.19  184.73  153.69  157.22  176.82  160.75  151.16  152.89  145.27  152.62  154.14  155.74  159.10  149.36  145.34  153.53  146.59  149.49  149.36  150.23 | 2161.94  2073.84  2174.99  2176.96  2174.59  2048.35  2131.74  2173.14  2172.50  2115.64  2172.24  1815.52  2040.49  2150.30  2194.19  1994.24  2163.86  2106.52  2081.99  2028.61  2121.61  2151.09  2173.58  2168.27  2084.57  2028.78  2139.40  2038.21  2078.63  2084.56  2088.89 | 2369.52 |
|  |  |  |  |  |  |  |  | 2290.35 |
|  |  |  |  |  |  |  |  | 2389.33 |
|  |  |  |  |  |  |  |  | 2386.41 |
|  |  |  |  |  |  |  |  | 2383.95 |
|  |  |  |  |  |  |  |  | 2258.28 |
|  |  |  |  |  |  |  |  | 2391.65 |
|  |  |  |  |  |  |  |  | 2389.16 |
|  |  |  |  |  |  |  |  | 2391.85 |
|  |  |  |  |  |  |  |  | 2391.56 |
|  |  |  |  |  |  |  |  | 2391.36 |
|  |  |  |  |  |  |  |  | 2013.85 |
|  |  |  |  |  |  |  |  | 2298.58 |
|  |  |  |  |  |  |  |  | 2356.58 |
|  |  |  |  |  |  |  |  | 2403.88 |
|  |  |  |  |  |  |  |  | 2242.84 |
|  |  |  |  |  |  |  |  | 2380.26 |
|  |  |  |  |  |  |  |  | 2310.94 |
|  |  |  |  |  |  |  |  | 2290.19 |
|  |  |  |  |  |  |  |  | 2227.57 |
|  |  |  |  |  |  |  |  | 2327.56 |
|  |  |  |  |  |  |  |  | 2358.02 |
|  |  |  |  |  |  |  |  | 2381.94 |
|  |  |  |  |  |  |  |  | 2381.97 |
|  |  |  |  |  |  |  |  | 2287.24 |
|  |  |  |  |  |  |  |  | 2227.84 |
|  |  |  |  |  |  |  |  | 2345.94 |
|  |  |  |  |  |  |  |  | 2238.76 |
|  |  |  |  |  |  |  |  | 2281.76 |
|  |  |  |  |  |  |  |  | 2287.22 |
|  |  |  |  |  |  |  |  | 2292.68 |
| **Average control** |  | **430.00** | **444.05** | **8.022** | **1933.27** | **158.06** | **2106.75** | **2321.58** |
| **Standard deviation** |  | **0.00** | **42.81** | **0.04** | **74.78** | **13.08** | **77.72** | **80.96** |
|  |  |  |  |  |  |  |  |  |
| **Treatment** | **Week** | **pCO2 aimed for**  **[ppm]** | **pCO2 obtained**  **[ppm]** | **pH** | **HCO_3_^-^**  **[µmol kg^-1^]** | **CO_3_^2-^**  **[µmol kg^-1^]** | **DIC**  **[µmol kg^-1^]** | **TA**  **[µmol kg^-1^]** |
| Elevated | 1 | 430 | 470.00 | 8.002 | 1972.40 | 153.64 | 2142.45 | 2349.03 |
|  | 2 | 600 | 653.00 | 7.871 | 2121.57 | 122.31 | 2267.73 | 2419.75 |
|  | 3 | 1000 | 1007.00 | 7.720 | 2199.34 | 89.61 | 2323.93 | 2417.83 |
|  | 4 | 1000 | 1022.00 | 7.715 | 2207.61 | 88.96 | 2332.07 | 2424.41 |
|  | 5 | 1000 | 1002.00 | 7.739 | 2129.94 | 90.58 | 2252.98 | 2351.94 |
|  | 6 | 1000 | 1000.00 | 7.728 | 2225.02 | 92.36 | 2352.12 | 2449.68 |
|  | 7 | 1000 | 1200.00 | 7.652 | 2240.13 | 78.01 | 2359.82 | 2430.17 |
|  | 8 | 1000 | 1100.00 | 7.692 | 2249.81 | 85.84 | 2373.87 | 2458.49 |
|  | 9 | 1000 | 1034.00 | 7.712 | 2216.02 | 88.60 | 2340.54 | 2431.82 |
|  | 10 | 1000 | 1012.00 | 7.728 | 2248.94 | 93.23 | 2377.33 | 2475.31 |
|  | 11 | 1000 | 1002.00 | 7.725 | 2214.04 | 91.26 | 2340.11 | 2436.26 |
|  | 12 | 1000 | 1100.00 | 7.689 | 2236.51 | 84.83 | 2359.55 | 2442.96 |
|  | 13 | 1000 | 1000.00 | 7.744 | 2307.99 | 99.37 | 2442.10 | 2548.03 |
|  | 14 | 1000 | 1090.00 | 7.701 | 2051.63 | 79.98 | 2165.71 | 2249.31 |
|  | 15 | 1000 | 1108.00 | 7.679 | 2202.56 | 81.68 | 2322.73 | 2401.96 |
|  | 16 | 1000 | 1171.00 | 7.668 | 2265.83 | 81.79 | 2388.30 | 2464.57 |
|  | 17 | 1000 | 1131.00 | 7.698 | 2059.74 | 79.81 | 2173.99 | 2256.87 |
|  | 18 | 1000 | 1111.00 | 7.684 | 2230.14 | 83.51 | 2352.24 | 2433.52 |
|  | 19 | 1000 | 1201.00 | 7.646 | 2212.42 | 76.03 | 2330.17 | 2398.09 |
|  | 20 | 1000 | 980.00 | 7.730 | 2188.66 | 91.19 | 2313.89 | 2411.11 |
|  | 21 | 1000 | 1000.00 | 7.731 | 2238.60 | 93.49 | 2366.82 | 2465.73 |
|  | 22 | 1000 | 1200.00 | 7.650 | 2231.19 | 77.39 | 2350.26 | 2419.86 |
|  | 23 | 1000 | 1090.00 | 7.693 | 2236.22 | 85.59 | 2359.67 | 2444.49 |
|  | 24 | 1000 | 1120.00 | 7.690 | 2279.72 | 86.57 | 2405.19 | 2489.68 |
|  | 25 | 1000 | 1200.00 | 7.639 | 2175.83 | 73.60 | 2291.12 | 2356.14 |
|  | 26 | 1000 | 1015.00 | 7.725 | 2240.35 | 92.25 | 2367.86 | 2464.50 |
|  | 27 | 1000 | 1002.00 | 7.728 | 2229.56 | 92.55 | 2356.91 | 2454.60 |
|  | 28 | 1000 | 800.00 | 7.808 | 2139.88 | 106.78 | 2274.45 | 2400.57 |
|  | 29 | 1000 | 1100.00 | 7.690 | 2238.85 | 85.01 | 2362.07 | 2445.69 |
|  | 30 | 1000 | 1100.00 | 7.696 | 2052.95 | 79.14 | 2166.60 | 2248.58 |
|  | 31 | 1000 | 980.00 | 7.743 | 2011.07 | 86.32 | 2127.76 | 2224.90 |
|  | 32 | 1000 | 1000.00 | 7.724 | 2018.72 | 82.97 | 2133.52 | 2224.24 |
| **Average elevated** |  | **969.6875** | **1031.28** | **7.720** | **2183.54** | **89.82** | **2308.56** | **2402.81** |
| **Standard deviation** |  | **5.44** | **87.11** | **0.07** | **84.88** | **14.73** | **84.29** | **79.75** |
|  |  |  |  |  |  |  |  |  |
| **Treatment** | **Week** | **pCO2 aimed for**  **[ppm]** | **pCO2 obtained**  **[ppm]** | **pH** | **HCO_3_**  **[µmol kg^-1^]** | **CO_3_**  **[µmol kg^-1^]** | **DIC**  **[µmol kg^-1^]** | **TA**  **[µmol kg^-1^]** |
| control fluctuating | 1 | 450 | 465.42 | 8.010 | 1979.44 | 197.94 | 157.05 | 2152.66 |
| control fluctuating | 2 | 460 | 487.69 | 8.008 | 2063.84 | 162.93 | 2243.71 | 2459.83 |
| control fluctuating | 3 | 480 | 475.00 | 8.011 | 2026.63 | 161.31 | 2204.44 | 2419.85 |
| control fluctuating | 4 | 400 | 453.25 | 8.001 | 1888.26 | 146.75 | 2050.76 | 2250.97 |
| control fluctuating | 5 | 570 | 598.76 | 7.891 | 1936.42 | 116.83 | 2074.05 | 2225.79 |
| control fluctuating | 6 | 450 | 472.11 | 8.001 | 1968.50 | 153.12 | 2138.01 | 2343.99 |
| control fluctuating | 7 | 450 | 474.50 | 8.000 | 1974.23 | 153.24 | 2143.95 | 2349.83 |
| control fluctuating | 8 | 460 | 469.98 | 8.005 | 1978.41 | 155.37 | 2150.10 | 2358.96 |
| control fluctuating | 9 | 430 | 433.00 | 8.014 | 1859.74 | 149.01 | 2023.79 | 2228.76 |
| control fluctuating | 10 | 500 | 472.85 | 8.003 | 1979.89 | 154.65 | 2150.97 | 2358.69 |
| control fluctuating | 11 | 400 | 430.00 | 8.049 | 2003.37 | 174.12 | 2192.43 | 2427.57 |
| control fluctuating | 12 | 630 | 650.16 | 7.904 | 2166.34 | 134.66 | 2323.58 | 2492.84 |
| control fluctuating | 13 | 520 | 532.70 | 7.940 | 1930.48 | 130.51 | 2079.50 | 2252.98 |
| control fluctuating | 14 | 440 | 452.38 | 8.029 | 2013.17 | 167.13 | 2196.02 | 2420.56 |
| control fluctuating | 15 | 450 | 467.00 | 8.018 | 2022.34 | 163.38 | 2201.93 | 2420.57 |
| control fluctuating | 16 | 400 | 446.56 | 8.017 | 1930.12 | 155.63 | 2101.26 | 2312.75 |
| control fluctuating | 17 | 500 | 538.31 | 7.937 | 1937.87 | 130.14 | 2086.71 | 2259.28 |
| control fluctuating | 18 | 500 | 510.00 | 7.995 | 2095.86 | 160.68 | 2274.26 | 2485.63 |
| control fluctuating | 19 | 480 | 489.44 | 7.974 | 1916.67 | 140.02 | 2073.70 | 2262.40 |
| control fluctuating | 20 | 430 | 440.00 | 8.026 | 1940.77 | 159.70 | 2115.76 | 2332.76 |
| control fluctuating | 21 | 460 | 474.49 | 7.980 | 1886.58 | 139.94 | 2043.00 | 2232.99 |
| control fluctuating | 22 | 400 | 428.99 | 8.025 | 1891.97 | 155.66 | 2062.53 | 2275.87 |
| control fluctuating | 23 | 500 | 478.00 | 8.026 | 2109.71 | 173.71 | 2300.03 | 2529.76 |
| control fluctuating | 24 | 560 | 566.21 | 7.945 | 2076.38 | 142.05 | 2238.10 | 2422.59 |
| control fluctuating | 25 | 590 | 561.74 | 7.938 | 2025.97 | 136.31 | 2181.80 | 2359.82 |
| control fluctuating | 26 | 420 | 420.00 | 8.051 | 1964.52 | 171.42 | 2150.53 | 2383.56 |
| control fluctuating | 27 | 630 | 620.89 | 7.898 | 2044.17 | 125.55 | 2191.29 | 2351.86 |
| control fluctuating | 28 | 480 | 498.34 | 7.961 | 1893.15 | 134.17 | 2044.63 | 2225.49 |
| control fluctuating | 29 | 420 | 435.00 | 8.041 | 1988.89 | 169.64 | 2173.64 | 2402.96 |
| control fluctuating | 30 | 460 | 460.00 | 7.997 | 1898.32 | 146.14 | 2060.44 | 2259.26 |
| control fluctuating | 31 | 500 | 510.00 | 7.995 | 2095.91 | 160.68 | 2274.31 | 2485.69 |
| control fluctuating | 32 | 480 | 497.96 | 7.961 | 1892.01 | 134.11 | 2043.41 | 2224.24 |
| **average control fluctuating** |  | **478.125** | **490.96** | **7.989** | **1980.62** | **151.77** | **2085.80** | **2342.84** |
| **standard deviation** |  | **62.14** | **96.85** | **0.04** | **176.73** | **17.13** | **361.56** | **97.54** |
|  |  |  |  |  |  |  |  |  |
| **Treatment** | **Week** | **pCO2 aimed for**  **[ppm]** | **pCO2 obtained**  **[ppm]** | **pH** | **HCO_3_^-^**  **[µmol kg^-1^]** | **CO_3_^2-^**  **[µmol kg^-1^]** | **DIC**  **[µmol kg^-1^]** | **TA**  **[µmol kg^-1^]** |
| elevated fluctuating | 1 | 430 | 456.46 | 8.069 | 1972.40 | 153.64 | 2142.45 | 2349.03 |
| elevated fluctuating | 2 | 653 | 633.80 | 7.908 | 2121.57 | 122.31 | 2267.73 | 2419.75 |
| elevated fluctuating | 3 | 870 | 947.19 | 7.789 | 2199.34 | 89.61 | 2323.93 | 2417.83 |
| elevated fluctuating | 4 | 1200 | 1266.23 | 7.668 | 2207.61 | 88.96 | 2332.07 | 2424.41 |
| elevated fluctuating | 5 | 1300 | 1158.52 | 7.630 | 2129.94 | 90.58 | 2252.98 | 2351.94 |
| elevated fluctuating | 6 | 1115 | 1266.09 | 7.685 | 2225.02 | 92.36 | 2352.12 | 2449.68 |
| elevated fluctuating | 7 | 936 | 968.23 | 7.768 | 2240.13 | 78.01 | 2359.82 | 2430.17 |
| elevated fluctuating | 8 | 840 | 879.13 | 7.898 | 2249.81 | 85.84 | 2373.87 | 2458.49 |
| elevated fluctuating | 9 | 1200 | 1199.95 | 7.652 | 2216.02 | 88.60 | 2340.54 | 2431.82 |
| elevated fluctuating | 10 | 1300 | 1276.32 | 7.615 | 2248.94 | 93.23 | 2377.33 | 2475.31 |
| elevated fluctuating | 11 | 718 | 725.85 | 7.851 | 2214.04 | 91.26 | 2340.11 | 2436.26 |
| elevated fluctuating | 12 | 1250 | 1313.70 | 7.626 | 2236.51 | 84.83 | 2359.55 | 2442.96 |
| elevated fluctuating | 13 | 1100 | 996.15 | 7.696 | 2307.99 | 99.37 | 2442.10 | 2548.03 |
| elevated fluctuating | 14 | 938 | 909.66 | 7.761 | 2051.63 | 79.98 | 2165.71 | 2249.31 |
| elevated fluctuating | 15 | 978 | 978.39 | 7.732 | 2202.56 | 81.68 | 2322.73 | 2401.96 |
| elevated fluctuating | 16 | 1400 | 1298.18 | 7.600 | 2265.83 | 81.79 | 2388.30 | 2464.57 |
| elevated fluctuating | 17 | 800 | 735.41 | 7.817 | 2059.74 | 79.81 | 2173.99 | 2256.87 |
| elevated fluctuating | 18 | 1060 | 1093.57 | 7.699 | 2230.14 | 83.51 | 2352.24 | 2433.52 |
| elevated fluctuating | 19 | 1200 | 1115.58 | 7.652 | 2212.42 | 76.03 | 2330.17 | 2398.09 |
| elevated fluctuating | 20 | 1200 | 1223.44 | 7.662 | 2188.66 | 91.19 | 2313.89 | 2411.11 |
| elevated fluctuating | 21 | 1300 | 1312.05 | 7.613 | 2238.60 | 93.49 | 2366.82 | 2465.73 |
| elevated fluctuating | 22 | 741 | 761.69 | 7.861 | 2231.19 | 77.39 | 2350.26 | 2419.86 |
| elevated fluctuating | 23 | 870 | 878.04 | 7.793 | 2236.22 | 85.59 | 2359.67 | 2444.49 |
| elevated fluctuating | 24 | 1304 | 1255.67 | 7.633 | 2279.72 | 86.57 | 2405.19 | 2489.68 |
| elevated fluctuating | 25 | 702 | 656.44 | 7.878 | 2175.83 | 73.60 | 2291.12 | 2356.14 |
| elevated fluctuating | 26 | 1077 | 1002.24 | 7.716 | 2240.35 | 92.25 | 2367.86 | 2464.50 |
| elevated fluctuating | 27 | 1200 | 1149.60 | 7.656 | 2229.56 | 92.55 | 2356.91 | 2454.60 |
| elevated fluctuating | 28 | 746 | 777.68 | 7.843 | 2139.88 | 106.78 | 2274.45 | 2400.57 |
| elevated fluctuating | 29 | 1500 | 1450.15 | 7.571 | 2238.85 | 85.01 | 2362.07 | 2445.69 |
| elevated fluctuating | 30 | 870 | 843.87 | 7.813 | 2052.95 | 79.14 | 2166.60 | 2248.58 |
| elevated fluctuating | 31 | 1100 | 1098.26 | 7.697 | 2011.07 | 86.32 | 2127.76 | 2224.90 |
| elevated fluctuating | 32 | 560 | 770.52 | 7.898 | 2018.72 | 82.97 | 2133.52 | 2224.24 |
| **average elevated fluctuating** |  | **985.46** | **1012.44** | **7.74** | **2183.54** | **89.82** | **2308.56** | **2402.81** |
| **standard deviation** |  | **262.41** | **244.48** | **0.12** | **86.24** | **14.96** | **85.64** | **81.02** |

SI Table 2: means and variances of ancestral and evolved plasticity. Mean evolved plasticity is higher in the fluctuating treatments than in the stable treatments. Additionally, variance increases in the stable and fluctuating controls, but it decreases in both elevated pCO_2_ treatments.

| Selection regime | Mean ancestral plasticity | Mean evolved plasticity | Variance evolved plasticity | Variance ancestral  plasticity |
| --- | --- | --- | --- | --- |
| SA | All 0.37 | 0.56 | 0.3 | All 0.06 |
| FA |  | 0.71 | 0.26 |  |
| SH |  | 0.44 | 0.02 |  |
| FH |  | 0.68 | 0.048 |  |

**Formulas for direct and correlated responses after evolution**

$$Direct response in growth to selection in the SH environment= \frac{\mu1000ppm CO2 assay after SH selection -\mu1000ppm CO2 assay in SA lineages}{\mu1000ppm CO2 assay in SA lineages}$$

$$Direct response in growth to selection in the FH environment= \frac{\mu1000ppm assay after FH selection -\mu1000ppm CO2 assay in FA lineages}{\mu1000ppm CO2 assay in FA lineages}$$

$$Correlated response in growth to selection in the SH environment= \frac{\mu430ppm assay after SH selection -\mu430ppm CO2 assay in SA selected lineages}{\mu430ppm CO2 assay in SA selected lineages}$$

$$Correlated response in growth to selection in the FH environment= \frac{\mu430ppm assay after FHselection -\mu430ppm CO2 assay in FA selected lineages}{\mu430ppm CO2 assay in FA selected lineages}$$

**Representative mean population sizes – we can exclude demographic effects**

**SA** 40694 ± 516 cells/ml

**FA** 40871 ± 709 cells/ml

**SH** 41008 ±631 cells/ml

**FH** 40999 ± 594 cells/ml

(No significant difference F _2,90_ = 1.21, p = 0.3)
